# Distinct prior expectations shape tactile and proprioceptive localization

**DOI:** 10.1371/journal.pone.0358656

**Published:** 2026-09-29

**Authors:** Hüseyin O. Elmas, W. Pieter Medendorp, Luke E. Miller

**Affiliations:** Donders Institute for Brain, Cognition and Behaviour, Radboud University, Nijmegen, The Netherlands; University of Hamburg, GERMANY

## Abstract

When a mosquito lands on your finger, swatting it away requires your brain to calculate its location in external space, which depends on the body’s 3D posture. Two competing hypotheses explain how the brain solves this challenge: *the integration hypothesis*, where tactile signals are transformed into spatial coordinates by integrating touch and posture information; and *the cueing hypothesis*, where touch merely cues a location on the body whose position is specified via proprioception. Adjudicating between these hypotheses is nearly impossible without modeling the latent factors underlying somatosensory spatial perception. We fill this gap in the present study. We first formalized each hypothesis from a Bayesian perspective: If touch merely triggers proprioceptive localization (cueing hypothesis), tactile and proprioceptive localization should rely on the same Bayesian computations, with identical prior expectations about the mosquito’s spatial location; If they involve distinct Bayesian computational processes (integration hypothesis), distinct prior expectations may shape tactile and proprioceptive localization. To test these predictions, we had nineteen participants localize either proprioceptive or tactile targets on their fingertips. We then fit their data with several Bayesian models of each hypothesis. Models allowing different prior distributions between modalities provided the best fit for most participants, with 15 out of 19 participants showing significantly different prior distributions across modalities. These provide strong computational evidence that tactile and proprioceptive localization rely on distinct computational mechanisms, a conclusion that has important implications for how we understand these everyday behaviors and their neural mechanisms.

## Introduction

Tactile localization is such a common everyday behavior that we hardly give it a second thought. Yet, the computational process underlying it remains a mystery. Picture a mosquito landing on your finger, which you want to swat away. The mosquito’s position is initially mapped in a skin-based reference frame that is implemented somatotopically in S1 [1]. However, simply knowing where the mosquito touches your skin is insufficient to swat it away, since movement shifts the same patch of skin to an entirely different position in space. How does the brain reliably locate tactile stimuli?

One prominent explanation for tactile localization, which we refer to as the *integration hypothesis*, proposes that tactile cues are combined with proprioceptive information about body posture [2]. This process remaps touch from the skin-based (somatotopic) to a spatial (spatiotopic) reference frame, often centered on another body part (e.g., eyes, trunk). There are several lines of evidence in favor of the integration hypothesis. For example, tactile localization judgments are affected by body posture, such as gaze direction [3,4]. Effects of body posture take several hundred milliseconds to influence a tactile decision [5–7], indicative of a dynamic process. Even non-spatial tasks, such as tactile temporal order judgments, are affected by task-irrelevant limb posture [8–11]. At a neural level, activity in frontal and parietal cortices represents tactile stimuli in external spatial coordinates [12–16]. Neurons in these regions often show multimodal tactile-proprioceptive receptive fields, responding to both tactile input and specific body positions [17–20].

An alternative proposal, the *reconstruction account*, suggests that tactile input only serves to indicate a location on the body part [21–24]. A recent study supporting this idea combined tactile localization with a temporal-order judgment task [23]. Participants pointed in external space to the first of two rapidly presented tactile stimuli, one on each hand. When participants incorrectly identified which hand was first touched, they tended to mislocalize the initial stimulus to the location of the second touched hand— rather than the first touched hand—at the time of the first stimulation. The authors argue that touch merely acts as a cue for a proprioceptive location of the body in space and is never remapped. Thus, according to the *reconstruction account*, localizing a touch on the fingertip is computationally equivalent to localizing the fingertip itself—suggesting that tactile localization operates similarly to proprioceptive localization.

In the present study, we use the term *cueing hypothesis* as an operational Bayesian formalization of this reconstruction account. In this formulation, tactile input specifies the contacted location on the body, whereas the external location assigned to that touch is derived primarily from a proprioceptive estimate of where the contacted body location is in space. Thus, a touch on the fingertip would serve as a cue to localize the fingertip via proprioception, similar to a verbal cue indicating which fingertip should be localized. If tactile and proprioceptive localization rely on the same underlying estimate of fingertip position, they should share the same systematic spatial bias structure.

Several studies have compared tactile and proprioceptive localization [25–28]. For example, Rincon-Gonzalez et al. [26] found that localizing touch on the hand preserved the overall spatial structure of estimation errors in proprioceptive hand localization, but the magnitude of errors was reduced. However, drawing strong theoretical inferences about the similarity of underlying mechanisms from behavioral data alone is challenging, as latent computational variables that distinguish between alternative mechanistic accounts—such as those posited above—are not immediately observable without computational modeling [29]. To this end, computational modeling may offer a promising approach to disentangle these sources of bias by relating observed behavior to underlying latent transformations and variables.

In the present study, we approach tactile and proprioceptive localization from a Bayesian perspective [30,31]. Bayesian models have proven effective for explaining the origins of perceptual errors and biases [32–35]. Because sensory input is noisy [36], the brain must make probabilistic inferences, combining uncertain sensory evidence with prior expectations [37]. Bayes’ theorem formalizes this process. In terms of localization, the probability of a particular stimulus location x given the sensory evidence (the posterior, P(x|e)), is proportional to the likelihood of observing that evidence (L(e|x)), multiplied by the prior probability of the stimulus (Π(x)); or, P(x|e)∝L(e|x)×Π(x). This approach reduces error by weighting evidence by its reliability, but it can also introduce systematic biases toward prior beliefs. Notably, Bayesian models have successfully explained several somatosensory phenomena, such as skin-based tactile localization [38], the cutaneous rabbit illusion [39,40], arm posture biases [41], and distorted body representations [42].

The cueing and integration hypotheses make competing predictions about the Bayesian computations involved in tactile and proprioceptive localization. The cueing hypothesis essentially equates tactile and proprioceptive localization, predicting they utilize the same prior expectations. In contrast, the integration hypothesis posits that localizing touch requires additional remapping transformations compared to localizing the body. These additional transformations may introduce different systematic biases. Moreover, the natural statistics encountered during tactile versus proprioceptive localization may differ, potentially leading to distinct prior expectations for each modality.

To directly adjudicate between these hypotheses, we designed a spatial localization experiment where participants either localized tactile targets on the fingers (tactile localization) or their fingers themselves (proprioceptive localization). Bayesian models were fit to localization errors in each task to test whether tactile and proprioceptive localization utilize the same (cueing hypothesis) or distinct priors (integration hypothesis). We found that models with significantly distinct spatial priors for tactile and proprioceptive localization provided better fits for most participants. These findings provide computational evidence against the cueing hypothesis and are in support of the integration hypothesis. In the following section, we first provide a brief, but more formal Bayesian description of the two hypotheses.

### Theoretical background

Consider the task of localizing a somatosensory target in a two-dimensional space. We can formalize this localization process as 2D Bayesian inference, where the perceived location (posterior, P) results from combining sensory measurements (likelihood, L) with prior expectations (Π):


$$\begin{array}{c} P\left(𝐱|𝐞\right)\propto L\left(𝐞|𝐱\right)\times \Pi \left(𝐱\right) \end{array}$$ (1)

Here 𝐱 denotes the 2D spatial location and 𝐞 the sensory measurements detected by proprioceptors and/or mechanoreceptors. L(𝐞|𝐱) represents the likelihood function that describes the probability of observing the sensory measurement given target at location 𝐱, and Π(𝐱) represents the expectations over possible target locations, derived from previous observations [43] and internal models [44]. The posterior distribution P(𝐱|𝐞) represents the probability distribution over possible stimulus locations given the sensory evidence; this computation is graphically depicted in Fig 1B.

**Fig 1:**
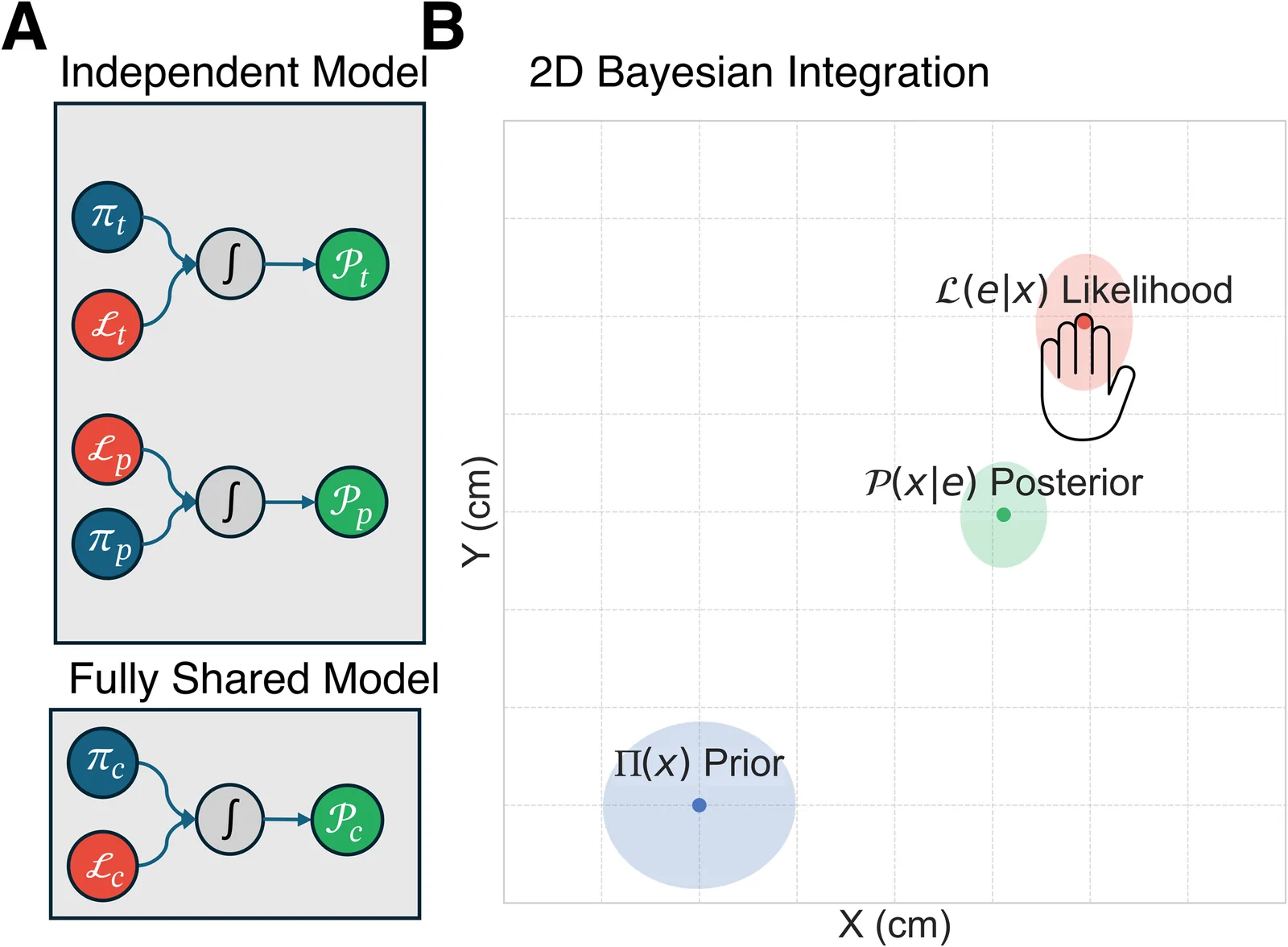
A) Comparison of model variants testing cueing vs. integration hypotheses. The Independent Model (top) allows separate priors ($\pi_{\text{t}}$, $\pi_{\text{p}}$) and likelihoods (${\text{L}}_{\text{t}}$, ${\text{L}}_{\text{p}}$) for tactile (t) and proprioceptive (p) conditions. The Fully Shared Model (bottom) uses identical priors ($\pi_{\text{c}}$) and likelihoods (${\text{L}}_{\text{c}}$). The arrows illustrate the computational flow: prior and likelihood are combined via integration (∫) to produce a posterior distribution (P). B) 2D Bayesian inference showing how spatial localization (posterior; green) emerges from combining prior expectations (blue) with sensory evidence (likelihood; red). As can be seen, the presence of a prior leads to a localization error (i.e., offset between likelihood and posterior).

Having introduced the general Bayesian framework, we can now formalize the integration and cueing hypotheses.

#### Integration hypothesis.

Consider the task of localizing in space the fingertip or a touch on the fingertip. According to the integration hypothesis, localizing proprioceptive and tactile targets involves distinct computational steps. Proprioceptively localizing the fingertip (${𝐱}_{p}$) requires mapping joint angles (θ) and body segment lengths (𝐥) to Cartesian coordinates. This can be expressed as:


$$\begin{array}{c} {𝐱}_{p}=f\left(\theta ,𝐥\right) \end{array}$$ (2)

where f is a transformation function (e.g., a kinematic chain function) that converts the internal body parameters into external spatial coordinates [41].

Localizing a tactile stimulus on the fingertip ${𝐱}_{t}$ requires additional transformations. Here, the mapping involves not only joint angles and segment lengths, but also a function g that maps the stimulus from somatotopic (skin-based) to spatiotopic Cartesian coordinates


$$\begin{array}{c} {𝐱}_{t}=f\left(\theta ,𝐥,g\left(𝐬\right)\right) \end{array}$$ (3)

As tactile localization also includes the needed proprioceptive transformations, the additional transformations g would likely introduce additional noise and/or biases to the resulting localization. In its strongest form, the integration hypothesis predicts distinct priors and likelihoods for tactile and proprioceptive localization, i.e., $\Pi_{t}\left(𝐱\right)\neq \Pi_{p}\left(𝐱\right)$ and likelihoods $L_{t}\left(𝐞|𝐱\right)\neq L_{p}\left(𝐞|𝐱\right)$.


$$\begin{array}{c} \begin{array}{cc} P_{t}\left(𝐱|𝐞\right) & \propto L_{t}\left(𝐞|𝐱\right)\times \Pi_{t}\left(𝐱\right)\\ P_{p}\left(𝐱|𝐞\right) & \propto L_{p}\left(𝐞|𝐱\right)\times \Pi_{p}\left(𝐱\right) \end{array} \end{array}$$ (4)

In a weaker version of the integration hypothesis, the tactile and proprioceptive likelihoods share the same uncertainty but still may have distinct spatial priors.

#### Cueing hypothesis.

In contrast, the cueing hypothesis proposes that tactile and proprioceptive localization rely on the same computational processes:


$$\begin{array}{c} {𝐱}_{t}={𝐱}_{p}=f\left(\theta ,𝐥\right) \end{array}$$ (5)

Here, tactile stimulation simply cues a specific body location, which is then localized using the same computational mechanisms underlying proprioceptive localization. Thus, the Bayesian inference underlying localization in both tasks involves common likelihoods and priors:


$$\begin{array}{c} \begin{array}{cc} P_{t}\left(𝐱|𝐞\right) & \propto L_{c}\left(𝐞|𝐱\right)\times \Pi_{c}\left(𝐱\right)\\ P_{p}\left(𝐱|𝐞\right) & \propto L_{c}\left(𝐞|𝐱\right)\times \Pi_{c}\left(𝐱\right) \end{array} \end{array}$$ (6)

In a weaker version of the cueing hypothesis, tactile and proprioceptive evidence has different levels of noise (e.g., added from the cueing)—and thus distinct likelihoods—while maintaining that both modalities access the same underlying spatial representation and thus share the same spatial prior.

## Methods

### Participants

The experiment initially included 21 healthy adults. Due to technical errors, data from two participants were excluded, leaving 19 participants (ages 17–23, 18 females) in the final analysis. All of them were right-handed, as assessed by the Edinburgh Handedness Inventory (score range: [62.5–100]), reported normal or corrected-to-normal vision, and had no history of neurological disorders. They provided written informed consent before the experiment, which was approved by the Ethics Committee of the Faculty of Social Sciences of the Radboud University. Participants were recruited between 21/10/2024 and 26/11/2024. Participants received course credits for their participation.

### Setup

Participants were seated in an adjustable chair, positioned at the edge of a table and facing the short side of a large computer monitor (42” LED iiyama ProLite TF4237MSC-B3AG, Tokyo, Japan, with dimensions 930 mm × 523 mm, 1920 × 1080 pixels). The computer monitor was placed on its backside in the lengthwise orientation, allowing participants to make judgments in depth. The monitor was securely placed on top of a table with a supportive structure (3 cm from the edge and 24 cm above the table). Participants were positioned with their body midline aligned to the screen center using a reference line displayed at the onset of a block of trials.

The participant’s left hand was placed under the computer monitor and on top of a stimulation platform embedded with three tactile solenoids. The solenoids were arranged to contact with the fingertips of the participant’s index, middle, and ring fingers. The solenoids were placed in a custom-made holder that maintained their stability and upright orientation. This holder was positioned between the monitor and the table surface (~20 cm below the screen). To ensure alignment between the screen coordinates and solenoid positions, we implemented a system using two perpendicular sliding rulers along horizontal and vertical dimensions. These rulers were aligned with the screen and positioned the solenoid holder directly beneath the screen with millimeter precision. The rulers could be locked at any location to prevent movement of the solenoid platform. During the experiment, participants used their right hand to respond with the mouse while their left hand rested on the solenoids.

To eliminate auditory cues from the tactile stimulators, participants wore noise-cancelling headphones (Sony WH-1000XM5) that delivered continuous white noise. Additionally, to eliminate visual feedback of the forelimbs, an opaque cloth covered participants’ hands, forearms, and the stimulation apparatus.

### Experimental design and procedure

In separate blocks of trials, participants performed either a tactile or proprioceptive localization task. In each trial of the respective task (Fig 2A), participants received either a tactile target (three brief taps, each lasting approximately 50ms, separated by short intervals, for a total sequence of roughly 0.5 s) or a proprioceptive target, which was indicated by text on the screen specifying one of the fingertips (presentation time: 1 s). Participants indicated the perceived target location on the screen using a mouse pointer, controlled with their right hand. Following target presentation, they had 5 s to respond. If they failed to respond within this period, a ‘Missed’ message appeared briefly on the screen (0.5 s), and the trial was recorded as a miss. Missed trials (~0.1% of all trials) were excluded from subsequent analyses. No visual feedback about localization accuracy was provided. Trials were separated by a 0.5 s inter-trial interval. During this period, the mouse cursor was invisible. At the start of each trial, the cursor reappeared at a random location within a 5 cm radius circle centered in the lower portion of the screen.

**Fig 2:**
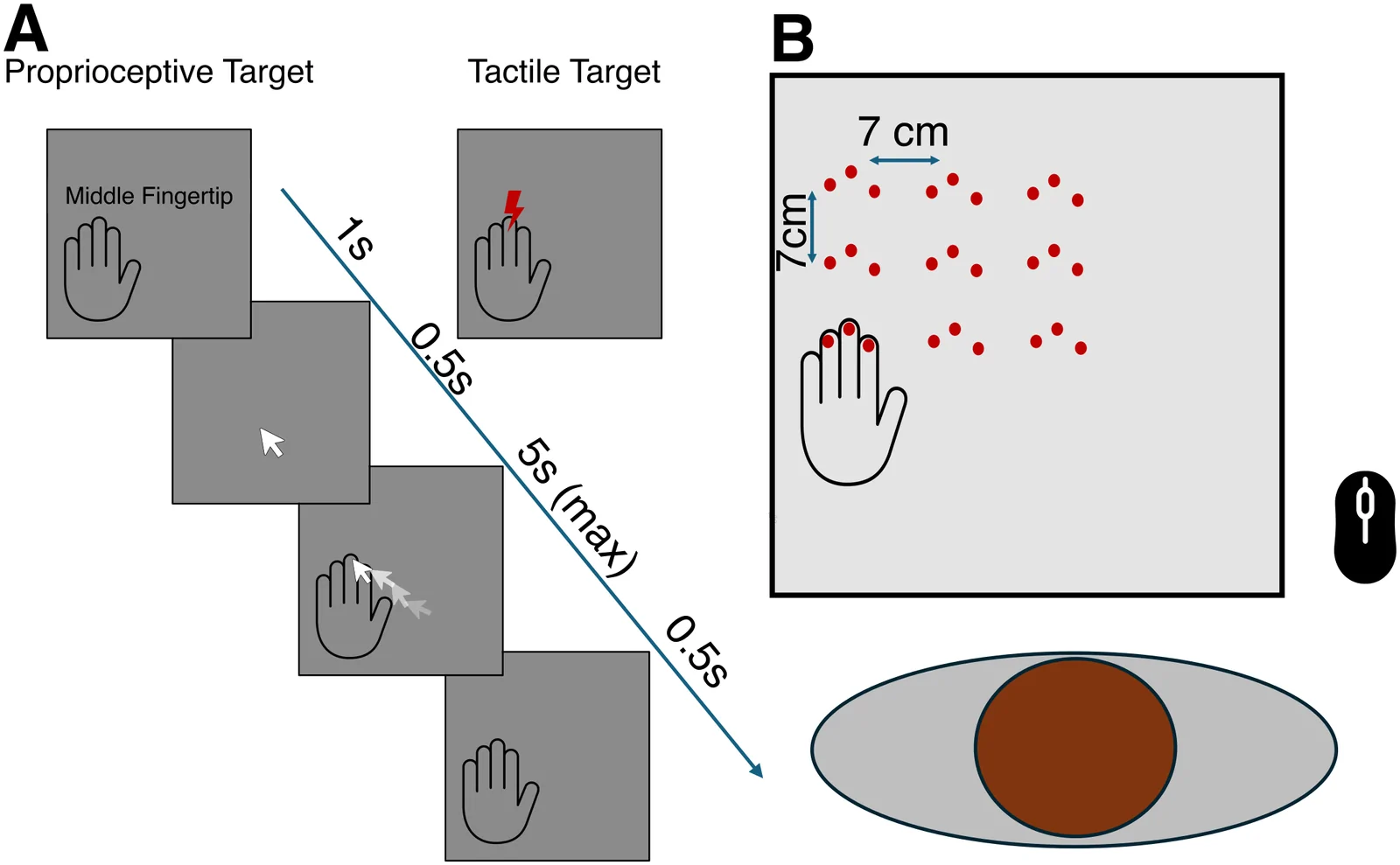
A) Trial structure showing temporal sequence for both tasks. Each trial began with the target presentation (0.5s): tactile stimulation or visual text indicating which fingertip to localize. Participants respond by clicking perceived target location on screen (5s max), followed by 0.5s inter-trial interval. B) Setup with participant positioned at horizontal monitor. The left hand was placed on a platform beneath the screen with fingertips contacting the solenoids. Red dots show all solenoid positions on the 3 × 3 grid of hand positions used across blocks.

The experiment comprised 18 blocks of 36 trials, with 9 blocks of tactile localization and 9 blocks of proprioceptive localization, presented in an intermingled order. Hand position varied across a pre-determined 3 × 3 grid with 9 different locations (Fig 2B), with a spacing of 7 cm between adjacent positions in both horizontal and vertical dimensions; the rightmost column of the grid was aligned approximately with the participant’s midline. In total there were 27 unique target locations (i.e., 3 fingers and 9 hand locations). Block order was randomized for each participant using a random permutation of the 18 blocks. Between blocks, participants could take breaks to prevent fatigue while the experimenter repositioned their left hand to the next location. Each block comprised 36 trials (12 trials per finger), with target fingers randomized within blocks. In total there were 108 trials per finger per task, across different hand locations (324 trials per task; 648 trials in total).

From the perspective of the cueing hypothesis, both tasks involved proprioceptive localization but with different cues: either a tactile cue, or a verbally written cue. In contrast, from the perspective of the integration hypothesis, the participants localized tactile targets and proprioceptive targets within their respective modalities.

### Behavioral analysis

To assess performance within each task, and to descriptively analyze the data, localization judgments on each trial were transformed into an error vector [26,45]. Error vectors were defined per trial as magnitude and direction of the response location versus the target location in the 2D plane. For each of the 27 target locations, and separately per task (tactile, proprioceptive), we calculated the mean ± SD of the magnitude and the circular mean ± circular SD of the direction across trials. For magnitude differences, we performed paired-samples t-tests on target-wise means to assess systematic accuracy differences between modalities. For angular distances, we calculated the absolute circular difference between mean error directions for each participant, then used Watson–Williams tests [46] to assess directional differences. Angular distances range from 0° (identical directions) to 180° (opposite directions). Finally, all trials were pooled across participants to report the grand-mean and SD of error magnitude and circular direction across all target locations.

### Computational modeling

#### Bayesian modeling framework.

To quantitatively distinguish between the cueing and integration hypotheses, we modeled participants’ localization responses as the output of a process of Bayesian inference (see Theoretical Background). In our computational modeling, we represented all spatial locations and responses in Cartesian coordinates within the 2D workspace. This modeling approach allowed us to decompose localization behavior into its underlying computational components: sensory uncertainty (likelihood) and spatial expectations (priors), enabling direct comparison of these parameters between tactile and proprioceptive conditions.

Spatial localisation for each task was formalised as 2D Bayesian inference in Cartesian coordinates,


$$\begin{array}{c} L_{m}\left(𝐞|𝐱\right)=N\left({𝐱}^{*},\Sigma_{lik}^{m}\right)\;\Pi_{m}\left(𝐱\right)=N\left(\mu_{pr}^{m},\Sigma_{pr}^{m}\right) \end{array}$$ (7)

where m∈{t,p,c} denote modality‑specific or shared parameters t (tactile), p (proprioceptive) and c (common) to both. ${𝐱}^{*}$ is a 2D vector, representing the true target location, $\Sigma_{lik}^{m}$ is a 2x2 matrix, capturing the sensory uncertainty and $\mu_{pr}^{m}$ and $\Sigma_{pr}^{m}$ represent likewise the mean and the uncertainty of the prior respectively. Note that we assume that the somatosensory system is well-calibrated to all measurements, and thus the likelihoods are centered on the true location $\left({𝐱}^{*}=𝐱\right)$. For each model variant and modality, the posterior was calculated according to Bayes’ rule:


$$\begin{array}{c} \mu_{\text{pos}}^{m}=\Sigma_{\text{pos}}^{m}\left({\left(\Sigma_{\text{pr}}^{m}\right)}^{-\text{1}}\mu_{\text{pr}}^{m}+{\left(\Sigma_{\text{lik}}^{m}\right)}^{-\text{1}}{𝐱}^{*}\right) \end{array}$$ (8)

$$\begin{array}{c} \Sigma_{\text{pos}}^{m}={\left({\left(\Sigma_{\text{pr}}^{m}\right)}^{-\text{1}}+{\left(\Sigma_{\text{lik}}^{m}\right)}^{-\text{1}}\right)}^{-\text{1}} \end{array}$$ (9)

Within the Bayesian modeling framework above, we developed four model variants with different parameter sharing structures to test the competing hypotheses:

*Independent Model* represents the strongest version of the integration hypothesis, where all likelihoods and priors are modality-specific:$\mu_{\text{pr}}^{t},\mu_{\text{pr}}^{p};\Sigma_{\text{pr}}^{t},\Sigma_{\text{pr}}^{p};\Sigma_{\text{lik}}^{t},\Sigma_{\text{lik}}^{p}$

*Shared Likelihood Model* represents a weaker version of the integration hypothesis, constraining likelihood uncertainty, $\Sigma_{\text{lik}}^{c}$, to be identical across modalities while retaining modality-specific priors: $\mu_{\text{pr}}^{t},\mu_{\text{pr}}^{p},\Sigma_{\text{pr}}^{t},\Sigma_{\text{pr}}^{p}$.

*Fully Shared Model* represents the strongest version of the cueing hypothesis, where the prior and likelihood are fully modality independent,$\mu_{\text{pr}}^{c},\Sigma_{\text{pr}}^{c},\Sigma_{\text{lik}}^{c}$

*Shared Prior Model* Represents a weaker version of the cueing hypothesis, imposing a common spatial prior, $\mu_{\text{pr}}^{c},\Sigma_{\text{pr}}^{c}$, but allowing modality-specific likelihood uncertainties$\Sigma_{\text{lik}}^{t},\Sigma_{\text{lik}}^{p}$

### Model fitting

To fit our models to the participant responses, we calculated the response distribution, which represents the probability of observing a response given a target location. The response distribution has the same mean with the posterior distribution, and its covariance can be calculated by:


$$\begin{array}{c} \begin{array}{cc} \Sigma_{r} & =W\Sigma_{\text{lik}}W^{T}\\ \text{where }W & ={\left(\Sigma_{\text{pr}}^{-\text{1}}+\Sigma_{\text{lik}}^{-\text{1}}\right)}^{-\text{1}}\Sigma_{\text{lik}}^{-\text{1}} \end{array} \end{array}$$ (10)

This reflects the fact that behavioral variability stems from the sensory noise and the likelihood’s weight relative to the prior.

We used maximum likelihood estimation to fit each model variant to the individual participant data. For each participant, the log-likelihood was computed as:


$$\begin{array}{c} LL=\sum_{i=\text{1}}^{N}\left[-\frac{k}{\text{2}}\text{log}(\text{2}\pi )-\frac{\text{1}}{\text{2}}\text{log}\left|\Sigma_{r}\right|-\frac{\text{1}}{\text{2}}{\left({obs}_{i}-\mu_{r}\right)}^{⊤}{\Sigma_{r}}^{-\text{1}}\left({obs}_{i}-\mu_{r}\right)\right] \end{array}$$ (11)

where N is the number of observations, $\mu_{r}$ is the mean and $\Sigma_{r}$ is the covariance of the response distribution, k is the number of dimensions (2) and ${obs}_{i}$ is the response location for trial i. Model fitting was performed separately for each participant using the full set of trial-level 2D responses. We did not fit the models to group-level averages or target-wise summary statistics. This allowed model comparison to be driven by each participant’s spatial response pattern across the workspace.

To avoid local minima, we used multi-start optimization with 100 random initial parameter sets for each model. Initial parameters were generated using Latin Hypercube Sampling to ensure efficient coverage of the parameter space. Parameter bounds were set to ensure valid covariance matrices and reasonable spatial scales over the workspace. The optimization was done using a custom script using SciPy in Python with L-BFGS-B algorithm [47].

### Model comparison

To determine which model best accounts for the observed spatial localization behavior, we compared models based on the Bayesian Information Criterion (BIC):


$$\begin{array}{c} BIC=-\text{2}LL+k\text{log}(n) \end{array}$$ (12)

where LL represents Log likelihood value, k is the number of free parameters, and n is the number of observations.

Next, we computed McFadden’s pseudo-$R^{\text{2}}$ [48] and Cox-Snell-$R^{\text{2}}$ [49] to assess model fit quality relative to a null model, where the null model that assumes responses were generated from a single bivariate Gaussian distribution that ignored both target locations and modality differences:


$$\begin{array}{c} p(obs)=N\left(obs|\mu_{\text{null}},\Sigma_{\text{null}}\right) \end{array}$$ (13)

where $\mu_{\text{null}}$ represents the global mean response location and $\Sigma_{\text{null}}$ represents the overall response covariance across all conditions. This model has five free parameters: two for the mean location ($\mu_{x}$, $\mu_{y}$) and three for the symmetric covariance matrix ($\sigma_{x}$, $\sigma_{y}$, ρ). The log-likelihood of this null model was computed as:


$$\begin{array}{c} {LL}_{\text{0}}=\sum_{i=\text{1}}^{N}\text{log}p\left({obs}_{i}|\mu_{\text{null}},\Sigma_{\text{null}}\right) \end{array}$$ (14)

where ${obs}_{i}$ represents the response location on trial i.

### Comparing priors

To quantitatively assess differences between tactile and proprioceptive spatial priors, we employed the Wasserstein-2 distance metric [50]. This metric was chosen for its ability to capture differences between probability distributions while maintaining mathematical properties such as symmetry and proper metric behavior. In our case, since we modeled spatial distributions as bivariate Gaussians, we utilized the closed-form solution:


$$\begin{array}{c} W_{\text{2}}^{\text{2}}(P,Q)={\left|\mu_{P}-\mu_{Q}\right|}_{\text{2}}^{\text{2}}+\text{tr}\left(\Sigma_{P}+\Sigma_{Q}-\text{2}{\left(\Sigma_{P}^{\text{1}/\text{2}}\Sigma_{Q}\Sigma_{P}^{\text{1}/\text{2}}\right)}^{\text{1}/\text{2}}\right) \end{array}$$ (15)

where P and Q are two bi-variate normal distributions, $\mu_{P}$, $\mu_{Q}$ represent the means and $\Sigma_{P}$ and $\Sigma_{Q}$ represent the covariance matrices of these distributions. The Wasserstein-2 distances reported below were obtained by taking the square root of Eq 15.

To determine statistical significance of observed distances between modality-specific distributions, we implemented a permutation test. For each participant, we computed the true Wasserstein-2 distance between fitted tactile and proprioceptive prior distributions from the Independent Model. We then generated a null distribution by repeatedly shuffling (1000 permutations) modality labels across trials and refitting the model to calculate resulting distances. Model fitting for the permutation analysis employed the same optimization framework as the main analysis (see above), with only 50 random starts per shuffle (instead of 100) to balance computational efficiency. If both modalities share the same underlying spatial representation, randomly reassigning trial labels should produce distances similar to the observed distance. In contrast, a significantly larger observed distance would indicate non-trivially distinct spatial representations for tactile and proprioceptive localization. Statistical significance was assessed by computing the proportion of null distances exceeding the observed distance, with significance threshold set at p < 0.05.

## Results

### Behavioral results – descriptive analysis

We first examined localization performance to assess whether tactile and proprioceptive conditions produced systematically different error patterns. Fig 3 displays the mean error vectors per participant and the group average for proprioceptive (red) and tactile (blue) task across the target locations. As shown, and consistent with prior findings [25–28], the magnitude and direction of errors differed between modalities at most locations.

**Fig 3:**
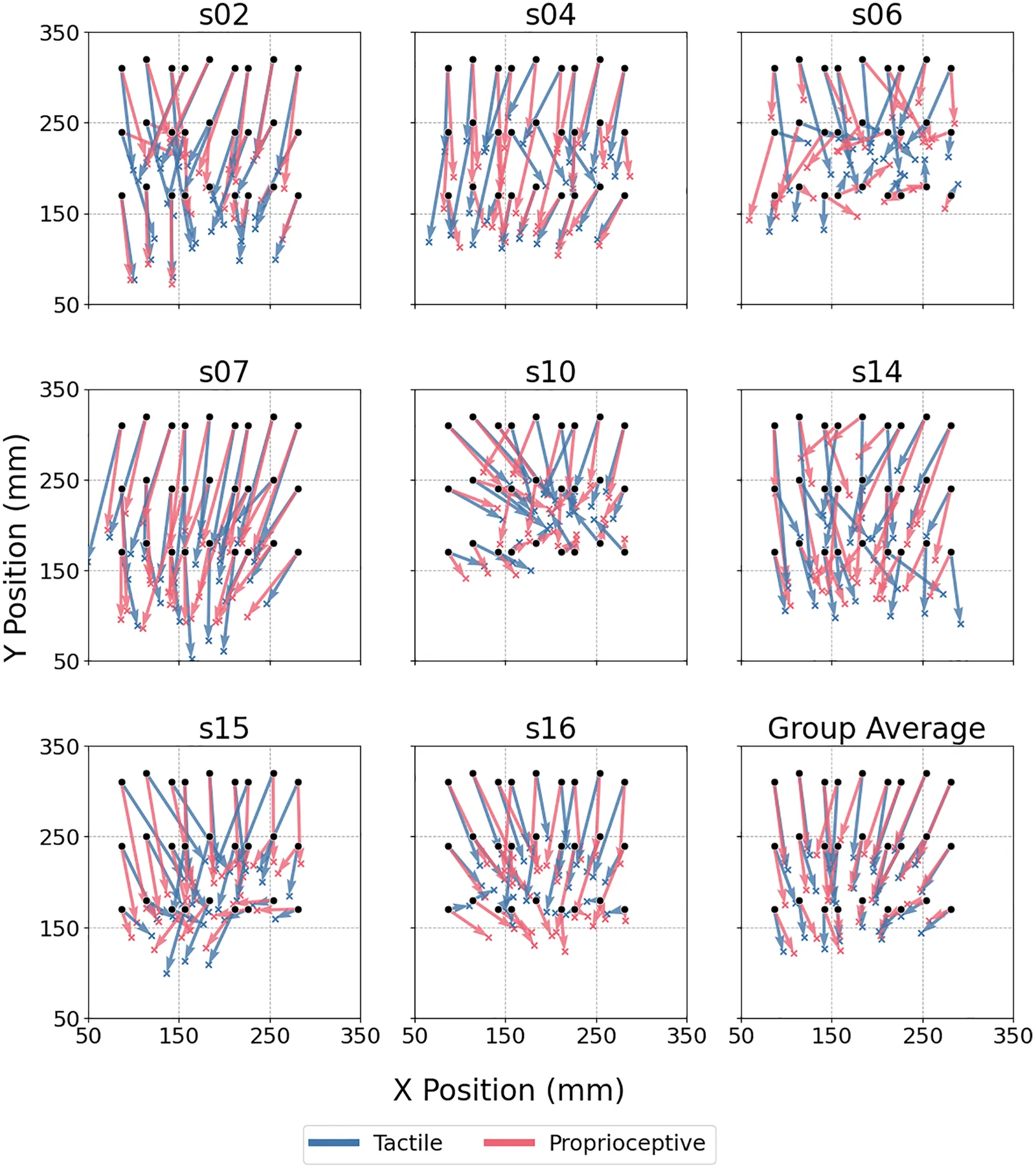
Mean error vectors for proprioceptive (red) and tactile (blue) localization, shown for representative individual participants with the group average in the final panel. Within each panel, arrows are drawn across all 27 target locations (three fingers × a 3 × 3 grid of hand positions); each arrow originates from a target position (black dots) and points toward the mean response location. Red arrows represent proprioceptive errors and blue arrows tactile errors.

Quantitatively, the mean between-modality difference in error magnitude was 2.0 mm (SD: 11.0 mm), with a mean directional difference of ${\text{1}\text{5}.\text{0}}^{\circ }$ (SD: ${\text{6}.\text{0}}^{\circ }$). Individual participant analyses revealed heterogeneous patterns of results: paired t-tests on error magnitudes identified significant modality differences in 8 of 19 participants (42.1%, p < 0.05), while Watson–Williams tests on error angles showed significant differences in 11 of 19 participants (57.9%, p < 0.05). While not all participants exhibited statistically detectable differences in both error components, a substantial proportion showed significant modality-specific patterns in either error magnitude or direction (16 of 19 participants (84%), showed at least one effect; Fig 4).

**Fig 4:**
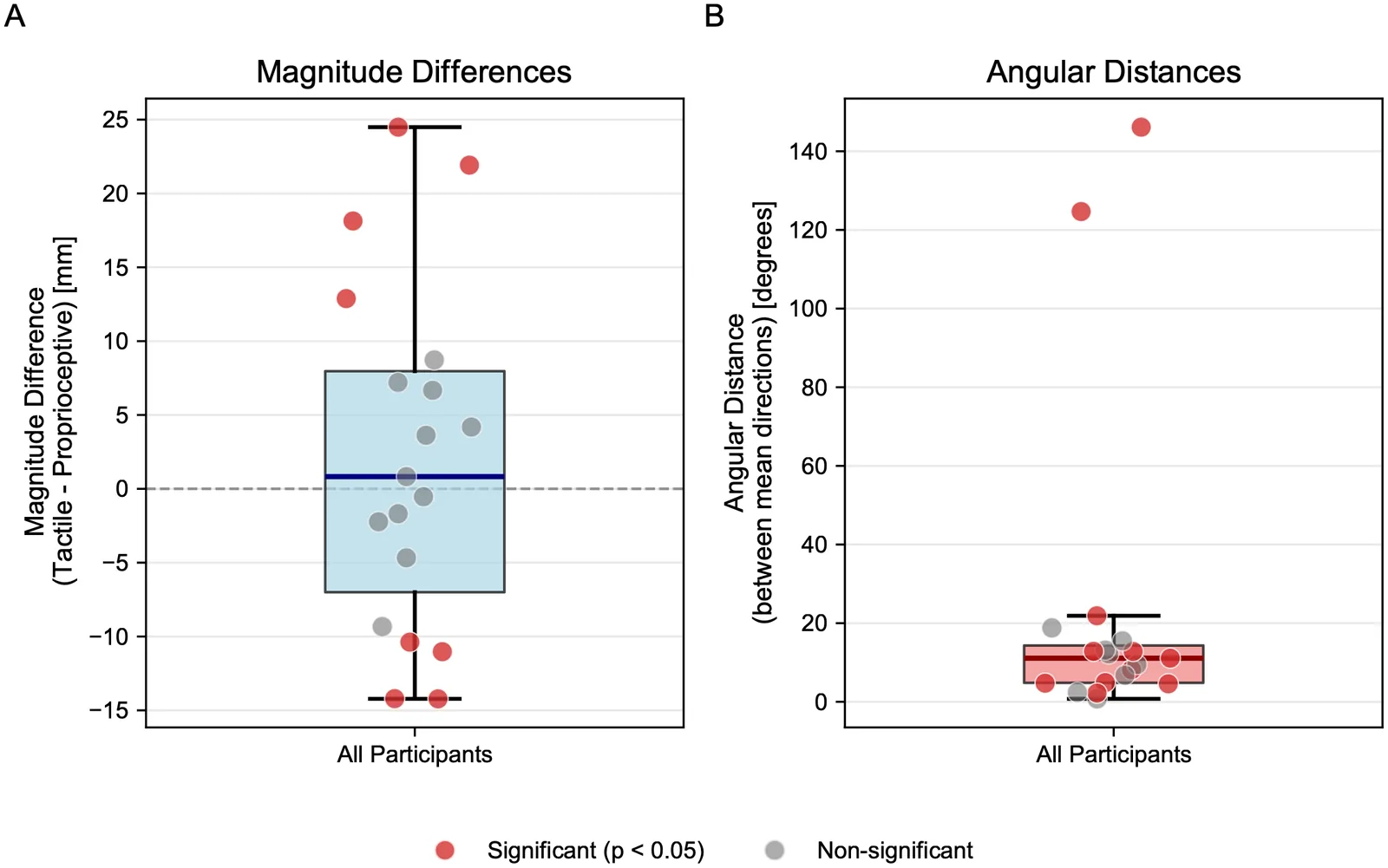
Differences in localization error across participants. A) Magnitude differences and B) angular distances between tactile and proprioceptive localization errors for individual participants. Angular distance represents the absolute circular difference between mean tactile and proprioceptive error directions for each participant. Red dots indicate significant differences (p < 0.05), gray dots indicate non-significant differences. Box plots show the distribution across all participants.

Although the group-level difference in error magnitude was small, the individual analyses did not indicate uniform similarity between tactile and proprioceptive localization. Participants varied in the size and direction of their modality effects. These results replicate previously observed differences in the error patterns between tactile and proprioceptive localization [25–28].

This participant-level heterogeneity motivated a modeling approach that preserved each participant’s spatial response pattern rather than reducing the data to a group-level error summary. Because descriptive group-level error measures cannot determine whether these participant-specific patterns reflect shared or modality-specific computational components, we applied Bayesian modeling to each participant’s localization behavior to identify the computational basis of these differences and to arbitrate between the cueing and integration hypotheses.

### Computational modeling results

We first evaluated model performance by comparing the predicted and observed response patterns across participants (Fig 5 and Fig 6). We systematically compared models representing different theoretical positions, beginning with the Independent and Fully Shared models as extreme versions of the integration and cueing hypotheses. The Independent Model outperformed the Fully Shared Model in 18 of 19 participants (mean ΔBIC: −210; range: [−425]; mean BF: $\text{4}.\text{5}\text{8}\times {\text{1}\text{0}}^{\text{4}\text{5}}$; Fig 7A). This is clear in Fig 5 and Fig 6, which compare the model predictions with the observed responses of several participants, for both tactile and proprioceptive localization respectively.

**Fig 5:**
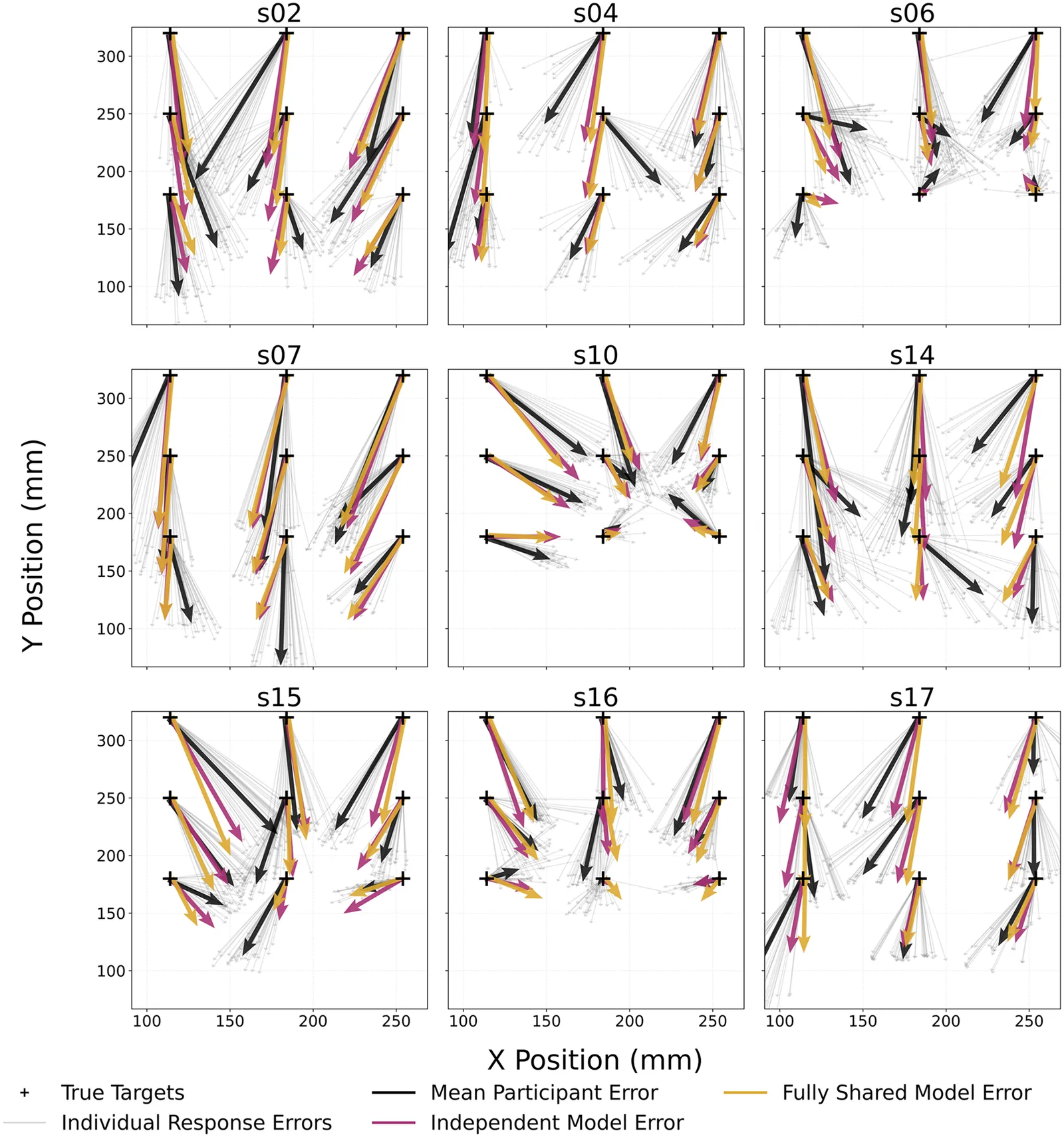
Tactile localization errors: Comparison of mean participant responses (black arrows) with Independent (magenta arrows) and Fully Shared Model (gold arrows) predictions across nine participants. For visualization clarity, responses from index and ring fingers have been spatially shifted and collapsed onto the middle finger position, allowing all 27 target locations and corresponding responses to be displayed in a 3x3 grid. The grid positions correspond to the 9 middle finger locations used in the experiment.

**Fig 6:**
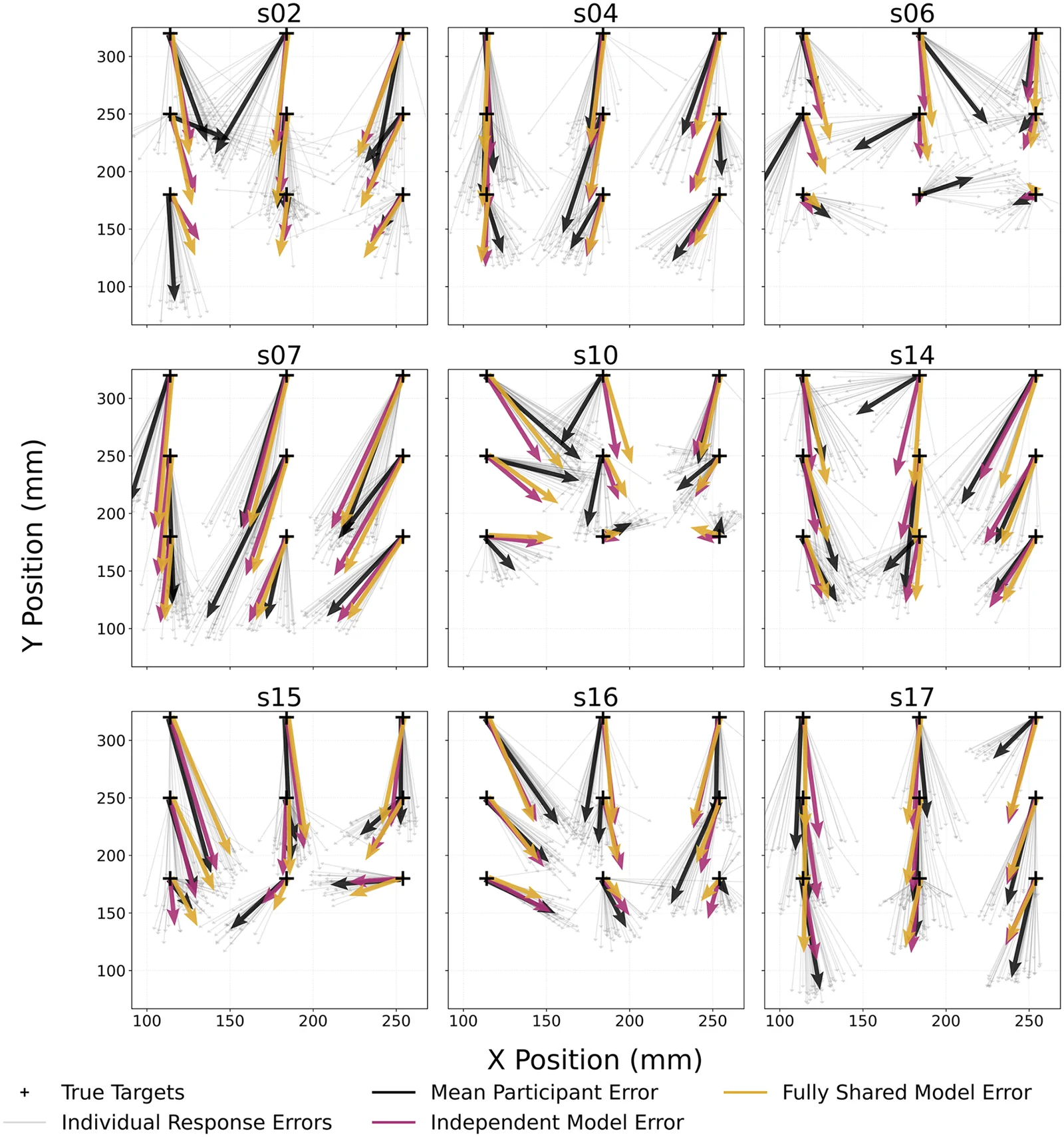
Proprioceptive localization errors: Comparison of mean participant responses (black arrows) with Independent (magenta arrows) and Fully Shared Model (gold arrows) predictions across nine participants. Data visualization is identical to Fig 5.

**Fig 7:**
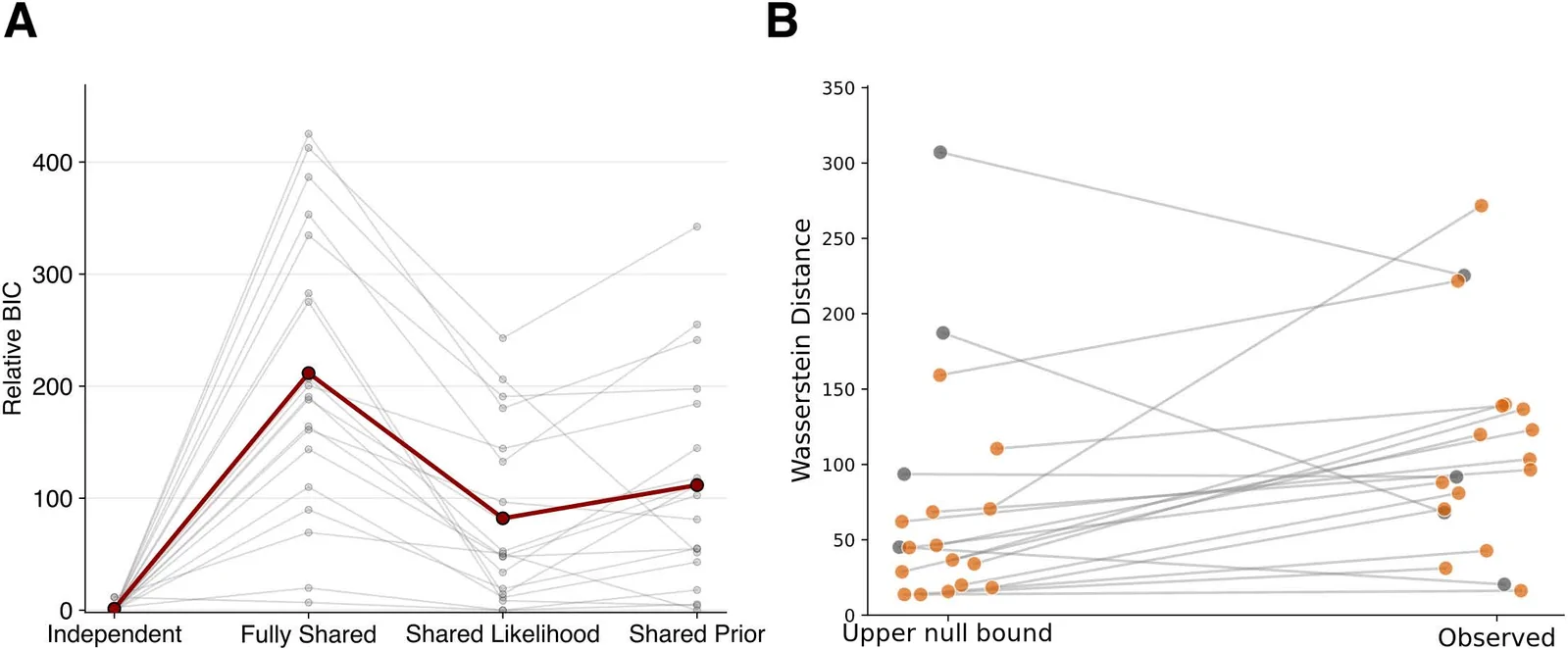
A) Model comparison across participants using Δ𝐁𝐈𝐂 relative to the best model for each participant. Each grey dot represents an individual participant’s ΔBIC for a given model, and the red dots represent the mean. B) Observed Wasserstein-2 distances versus the upper 95th percentile thresholds from null distributions for all participants (1000 shuffles per participant). Orange dots indicate participants with significantly different priors between modalities, whereas grey dots indicate participants with non-significant differences.

The Independent Model also outperformed the Shared Prior Model in 17 of 19 participants (mean ΔBIC:−110; range: [−342, 11]; mean BF: $\text{9}.\text{1}\text{9}\times {\text{1}\text{0}}^{\text{2}\text{3}}$), while the Shared Likelihood Model outperformed the Shared Prior Model in 15 of 19 participants (mean ΔBIC:−29; range: [−122, 154]; mean BF: $\text{2}.\text{7}\text{3}\times {\text{1}\text{0}}^{\text{6}}$). Among integration-supporting models, the Independent Model was superior to the Shared Likelihood Model in 17 of 19 participants (mean ΔBIC:−81; range: [−242, 11]; mean BF: $\text{3}.\text{3}\text{7}\times {\text{1}\text{0}}^{\text{1}\text{7}}$), favoring models with greater parameter independence between modalities.

Finally, we assessed the Independent Model’s overall explanatory power using multiple pseudo-$R^{\text{2}}$ measures relative to a null model. The Independent Model achieved moderate explanatory power across participants (McFadden’s pseudo-$R^{\text{2}}$: 0.15 ± 0.03; Cox-Snell pseudo-$R^{\text{2}}$: 0.43 ± 0.09). These likelihood-based pseudo-$R^{\text{2}}$ values suggest improvement over a null model that ignored target location and modality; they do not estimate the proportion of behavioral variance explained. Because pseudo-$R^{\text{2}}$ values are typically much lower than ordinary least-squares $R^{\text{2}}$ [51], with McFadden suggesting that values of 0.2–0.4 already indicate good model fit, the McFadden value of 0.15 should not be read as “15% of variance explained.”

A crucial distinction between the cueing and integration hypotheses is the nature of the prior expectations underlying tactile and proprioceptive localization. That is, the cueing hypothesis posits that priors are modality-independent, whereas the integration hypothesis suggests that they are modality specific. While the ΔBIC comparison suggested that the Independent Model provided better fit for most participants, this could also result from improved model fit estimation without substantially different priors. Fig 8 shows the modality-specific prior distributions over the workspace of example participants. These plots suggest differences in the spatial biases captured by the Independent Model for tactile and proprioceptive localization. Going beyond visual inspection, we therefore sought to quantitatively confirm that fits from the Independent Model captured statistically significant modality-specific priors, as this would be evidence against the cueing hypothesis. To examine whether the fitted spatial priors differed beyond what might be expected from random variation, we quantified the magnitude of differences between modality-specific priors using the Wasserstein-2 metric with permutation testing. Permutation testing of the distances between the modality-specific priors showed that 15 of 19 participants (78.9%) exhibited significant differences between tactile and proprioceptive prior distributions (p < 0.05; Fig 7B), suggesting that observed modality-specific spatial representations cannot be attributed to random variation.

**Fig 8:**
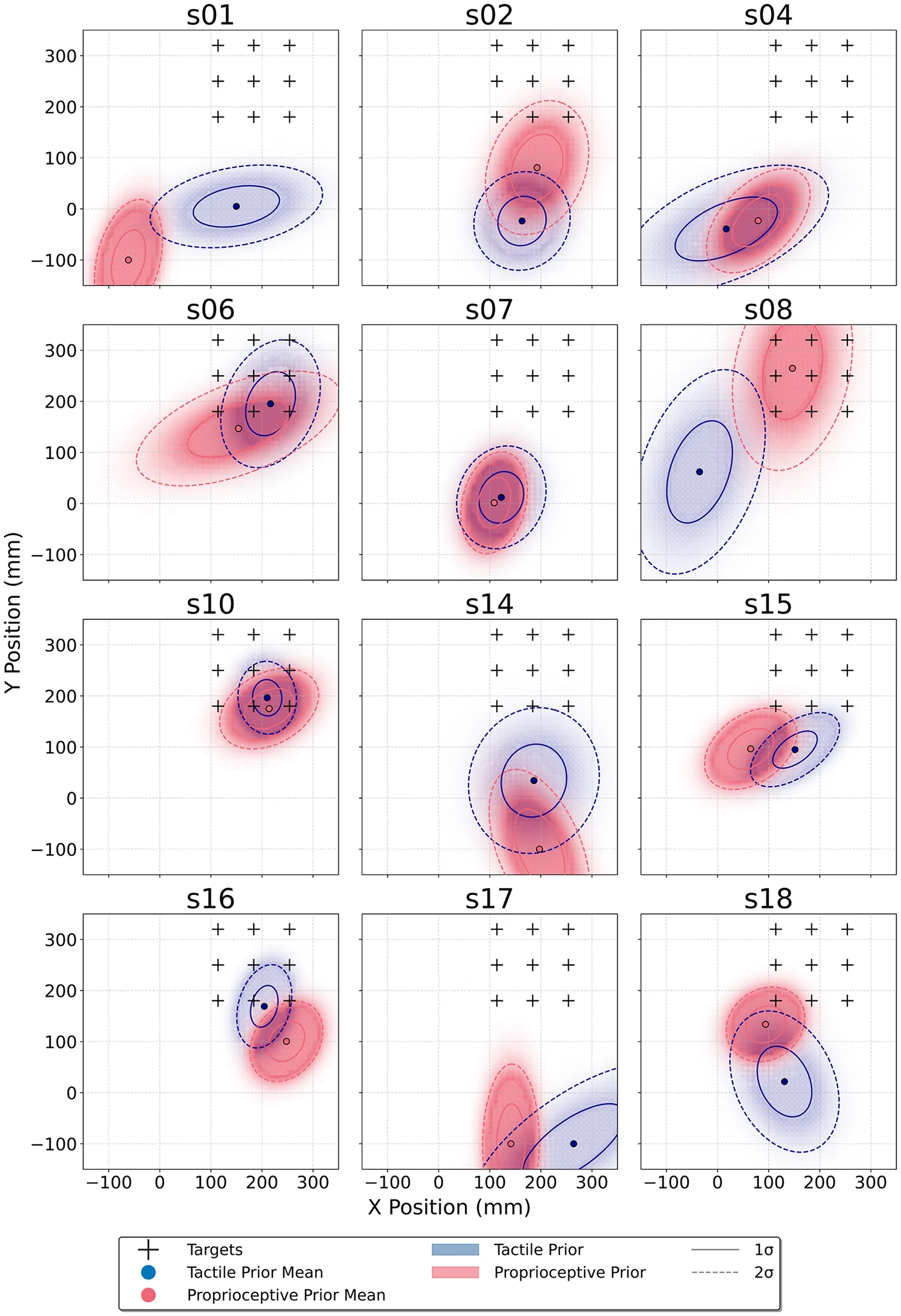
Fitted spatial priors from the Independent Model for representative participants. Each panel depicts one participant’s workspace where black crosses mark target locations. Colored regions represent 2D Gaussian prior distributions with their means: blue shading illustrates the covariance ellipse of the tactile priors; red shading illustrates the covariance ellipse of the proprioceptive priors.

As a robustness check, we repeated the model comparison and permutation analysis with prior means constrained to the physical screen workspace; the results were unchanged. A model-recovery analysis further showed high recovery accuracy across the constrained model family, indicating that the candidate models were distinguishable and that the preference for modality-specific priors was not driven by a generic bias toward the most flexible model. Together, these analyses indicate that tactile and proprioceptive localization were better explained by modality-specific priors, and that this conclusion did not depend on allowing prior means to fall outside the physical workspace (see S1 Text).

## Discussion

In this study, we combined psychophysics (tactile and proprioceptive localization in 2D space) and Bayesian modeling to determine the computational underpinnings of tactile localization. This approach allowed us to adjudicate between two competing hypotheses: the integration hypothesis [2], which proposes that tactile inputs are remapped into external spatial coordinates via integration with proprioception; and the cueing hypothesis [23]. Specifically, the integration hypothesis allows for distinct spatial priors, whereas the cueing hypothesis requires identical priors for tactile and proprioceptive localization. To differentiate between the two hypotheses, we implemented and compared four Bayesian model variants with different parameter-sharing structures, from fully independent parameters (supporting the strong integration hypothesis) to fully shared parameters (supporting the strong cueing hypothesis).

We found significant evidence in favor of the integration hypothesis, demonstrating that tactile and proprioceptive localization rely on distinct computational mechanisms. Indeed, the best-fitting model was the Independent Model, which was the most strongest version of the integration hypothesis. Crucially, most participants showed significantly different spatial prior distributions between tactile and proprioceptive localization, providing direct computational evidence against the cueing hypothesis. Our results challenge the view that tactile localization is computationally equivalent to proprioceptive localization [21], and instead suggest that tactile localization involves multimodal integration that remaps tactile inputs into external spatial coordinates. More broadly, these results suggest that somatosensory spatial localization involves multiple, specialized representational systems that maintain distinct computations, rather than relying on a single shared spatial framework.

An important distinction of our approach from other studies [3,5,23,25,26] was the use of Bayesian modeling to test competing hypotheses about tactile and proprioceptive localization mechanisms. While Bayesian modeling has been successfully applied to various aspects of sensory processing [33,35,52–54], its application to body representation and tactile-proprioceptive integration remains sparse [38,39,42]. By decomposing observed behavior into its computational components [30,31], we were able to gain insight into the spatial architecture of touch and proprioception that would not have been possible otherwise. Our study therefore highlights the theoretical benefits of using modeling to distinguish between competing hypotheses.

We observed considerable differences between the fitted priors for tactile and proprioceptive localization (Fig 7B, Fig 8). These modality-specific bias patterns may arise from several computational and contextual factors. One possibility is that tactile localization involves a different sequence of coordinate transformations than proprioceptive localization. While proprioceptive localization involves transforming joint angles and limb configurations directly into external spatial coordinates [41], tactile localization might first map touch from skin-based coordinates to limb-based representations [38]—only then is it possible to integrate touch with proprioceptive information to achieve external spatial localization [1,2,7,55]. Each additional transformation step could introduce systematic errors that accumulate, potentially leading to the distinct spatial biases that manifest as different prior distributions [56–58].

These fitted priors are best interpreted as latent spatial bias terms rather than as learned expectations about the workspace, since participants received no feedback about target locations or response accuracy. Consistent with this, constraining the priors to the physical workspace did not alter the preference for modality-specific priors, indicating that the modality difference reflects genuine bias structure rather than an artifact of unconstrained prior locations. Importantly, our theoretical inference depends on the relative structure of the fitted priors, not their absolute locations: within the same participants, workspace, response method, and modeling framework, tactile and proprioceptive localization were better explained by different prior structures. Because biases common to both tasks would be captured by a single shared prior, the preference for modality-specific priors suggests that the two tasks were shaped by partly distinct spatial biases.

Differences in environmental statistics could provide a contextual basis for distinct priors across different modalities, including vestibular [59], audition [60], and vision [61,62]. Given that tactile and proprioceptive localization often occur in different spatial contexts, it is reasonable to assume that they may also have distinct statistics. When tactile events occur on the fingertips, the hands are often positioned to interact with objects or explore surfaces. These functional positions may differ from typical resting arm configurations [43]. These differences in environmental statistics could favor distinct spatial priors. However, maintaining separate priors could entail computational costs in neural resources. The existence of modality-specific priors in our data suggests these costs may be outweighed by functional benefits for tactile localization.

Localizing a touch in external space involves several multimodal regions throughout frontal and parietal cortices [18,20,63]. The posterior parietal cortex (PPC) appears to be especially important in this ability [64] and has traditionally been thought to implement the remapping of tactile stimuli from skin-based coordinates into a spatiotopic representation [12–14,16,65]. Proponents of the cueing hypothesis would interpret these findings differently. Rather than a spatial representation of touch, activity within these regions reflects the coding of a specific location on the body. Indeed, localizing proprioceptive targets involves similar regions of PPC [66]. Though our study was only behavioral, our findings do have implications for how to interpret neural data. Specifically, given that our study provides evidence for tactile remapping, at least some frontal and parietal regions are involved in tactile localization above and beyond localizing the body.

This further raises the question of how the elements of our model might be implemented in the brain. We found that tactile and proprioceptive localization involved distinct sources of sensory evidence. This is consistent with what we know about the earliest sensory processing of both modalities. Touch and proprioception are only integrated once signals have reached Areas 1 and 2 of primary somatosensory cortex [1], at least 70 ms after sensory input [67]. Furthermore, our Bayesian modeling revealed that most participants exhibited significantly different spatial priors between tactile and proprioceptive conditions. These modality-specific spatial priors might be encoded as systematic differences in the preferred spatial locations of neural populations, with different tuning curve centers [68] or response patterns between tactile and proprioceptive networks [69,70]. The encoding of such priors is likely reflected in how the responses of neural populations vary across space. Recent findings show that tactile and proprioceptive space is represented by spatial gradients in overall response magnitude [71–73]. Priors could be implemented in biases of these gradients in the direction of spatial expectations.

While our results support the integration hypothesis over the cueing hypothesis, several limitations should be acknowledged. First, our modeling framework examined the outcomes of remapping processes in Cartesian space rather than the transformations themselves. Accordingly, the models were designed primarily to distinguish between competing hypotheses about parameter sharing, rather than to capture every source of participant-specific response bias. We did not measure joint angles or body segment lengths, which would have enabled direct modeling of the kinematic transformations from proprioceptive signals to spatial coordinates [41]. Furthermore, our Bayesian models assumed Gaussian distributions for both priors and likelihoods. While this assumption enabled tractable modeling, real neural representations likely involve more complex, non-Gaussian distributions (for instance due to signal-dependent noise in joint angles [74]). Future research should address these limitations through more naturalistic experimental paradigms utilizing virtual reality or multisensory environments, and computational modeling that explicitly characterizes the full kinematic chain from sensory input to spatial output.

To conclude, we provided computational evidence that tactile and proprioceptive localization rely on distinct spatial representations. By directly comparing each mode of localization, we were able to adjudicate between two competing hypotheses of how touch is localized in external space. Indeed, Bayesian modeling demonstrated that tactile and proprioceptive localization relied on distinct spatial expectations about stimulus location. This finding strongly suggests that localizing a touch on the body and localizing the body itself are not the same process, a conclusion that has important implications for how we understand these everyday behaviors and their neural mechanisms.

## Supporting information

S1 Text Supplementary robustness analyses. Robustness analyses for screen-space constrained priors. Fig A. Screen-space constrained model comparison. Fig B. Permutation test of screen-space constrained priors. Fig C. Model recovery for the screen-space constrained model family.
(PDF)
