# Supplementary material for: Distinct prior expectations shape tactile and proprioceptive localization
Source: PLoS One. 2026 Sep 29;21(9):e0358656. doi: 10.1371/journal.pone.0358656 (PMC13623134; doi:10.1371/journal.pone.0358656)
Supplement: Supplementary file 1 [file pone.0358656.s001.pdf]

S1 Text

Supplementary robustness analyses for screen-space constrained priors

Hüseyin O. Elmas W. Pieter Medendorp Luke E. Miller

Supplementary Robustness Analyses

Three robustness analyses assessed whether our main conclusion—that tactile and proprioceptive localization are better explained by modality-specific priors—depended on allowing fitted prior means to fall outside the response workspace or on a bias toward more flexible models. The first analysis repeated the model comparison within a screen-space constrained model family, in which prior means were restricted to the physical screen workspace (x: 0 to 523.26 mm; y: 0 to 930.24 mm), while the prior covariance, likelihood covariance, and correlation parameters were otherwise unconstrained. This constrained family comprised the same four models used in the main analysis: the Independent, Shared Likelihood, Shared Prior, and Fully Shared models.

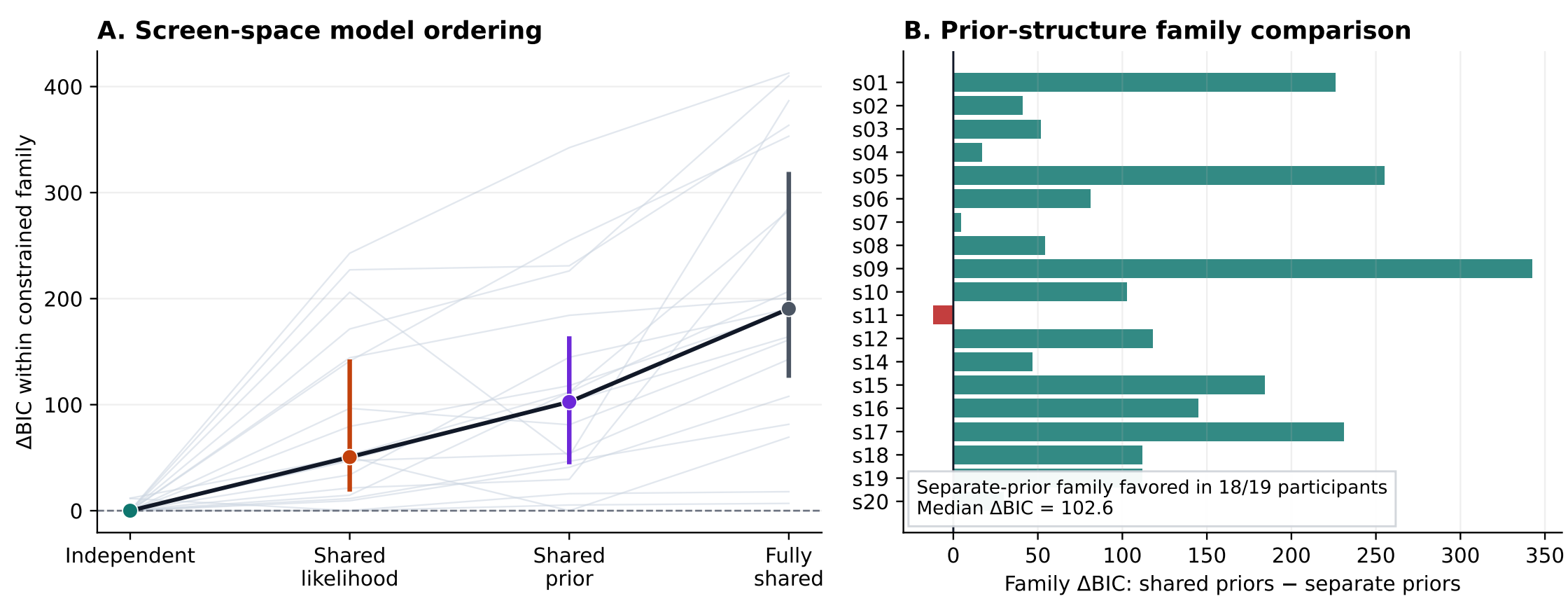

Fig A. Screen-space constrained model comparison. (A) BIC comparison within the screen-space constrained model family, plotted relative to the Independent Model. Values above zero indicate poorer fit than the Independent Model. The Independent Model remained the best-fitting model in 17 of 19 participants. (B) Family-level comparison of separate-prior and shared-prior models. Positive values indicate evidence favoring separate priors.

The separate-prior family was favored in 18 of 19 participants (median family-level ΔBIC = 102.58).

The second analysis repeated the permutation test using the screen-space constrained Independent Model. For each participant, modality labels were permuted across trials to generate a null distribution against which the observed tactile–proprioceptive prior distance was compared.

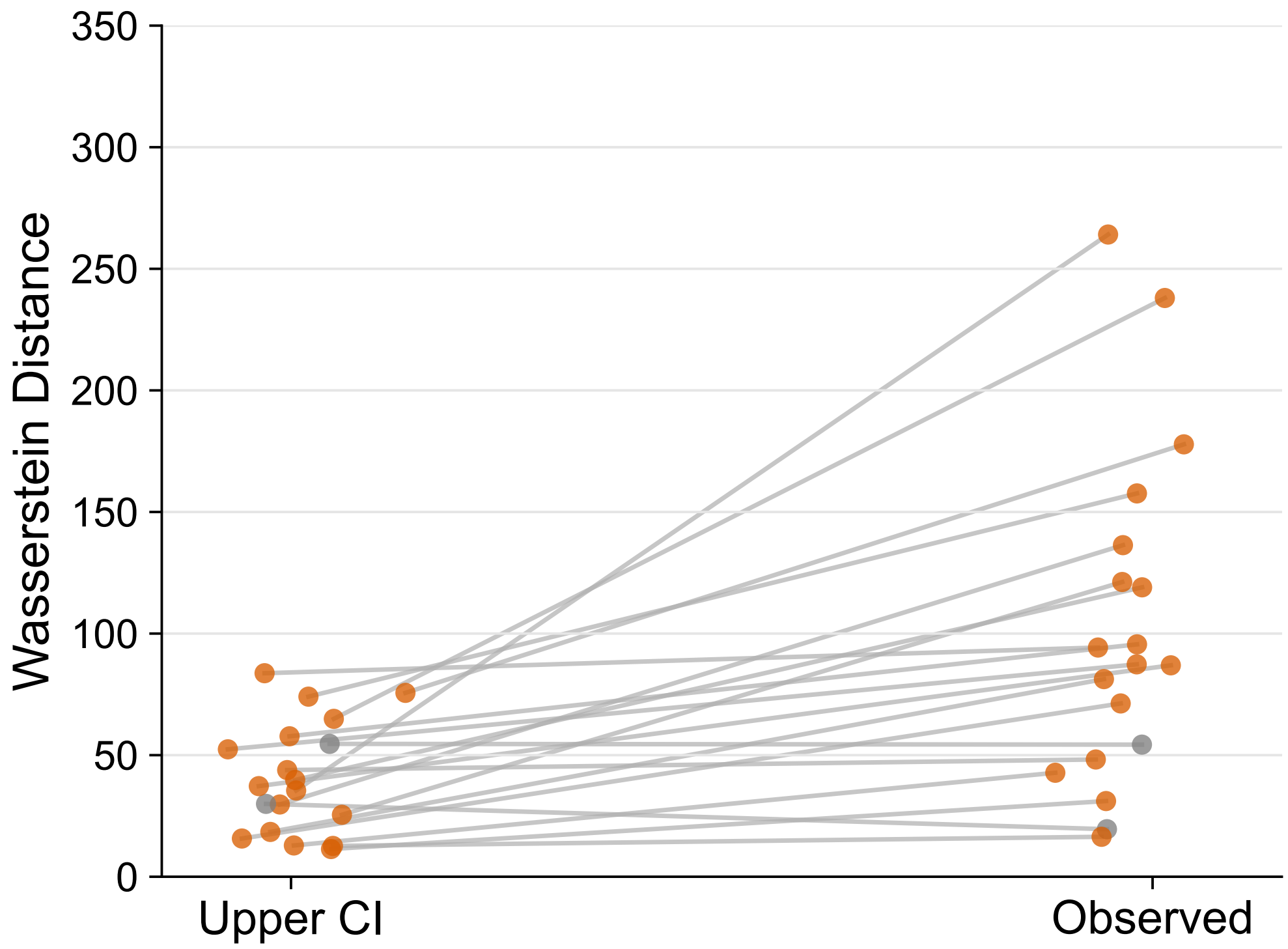

Fig B. Permutation test of screen-space constrained priors. Observed Wasserstein distances between tactile and proprioceptive fitted priors are plotted against the upper 95% threshold of the participant-specific permutation null distribution. Orange points indicate participants whose observed distance exceeded the null threshold; gray points indicate nonsignificant participants. The observed distance exceeded the null threshold in 17 of 19 participants, indicating that the modality-specific prior differences were not explained by random relabeling of task condition.

The third analysis was a model-recovery analysis assessing whether the constrained models were mutually distinguishable. For each participant and each constrained generating model, we simulated 50 datasets from the fitted parameters and refitted all four constrained candidate models, yielding 3,800 simulated datasets and 15,200 model fits.

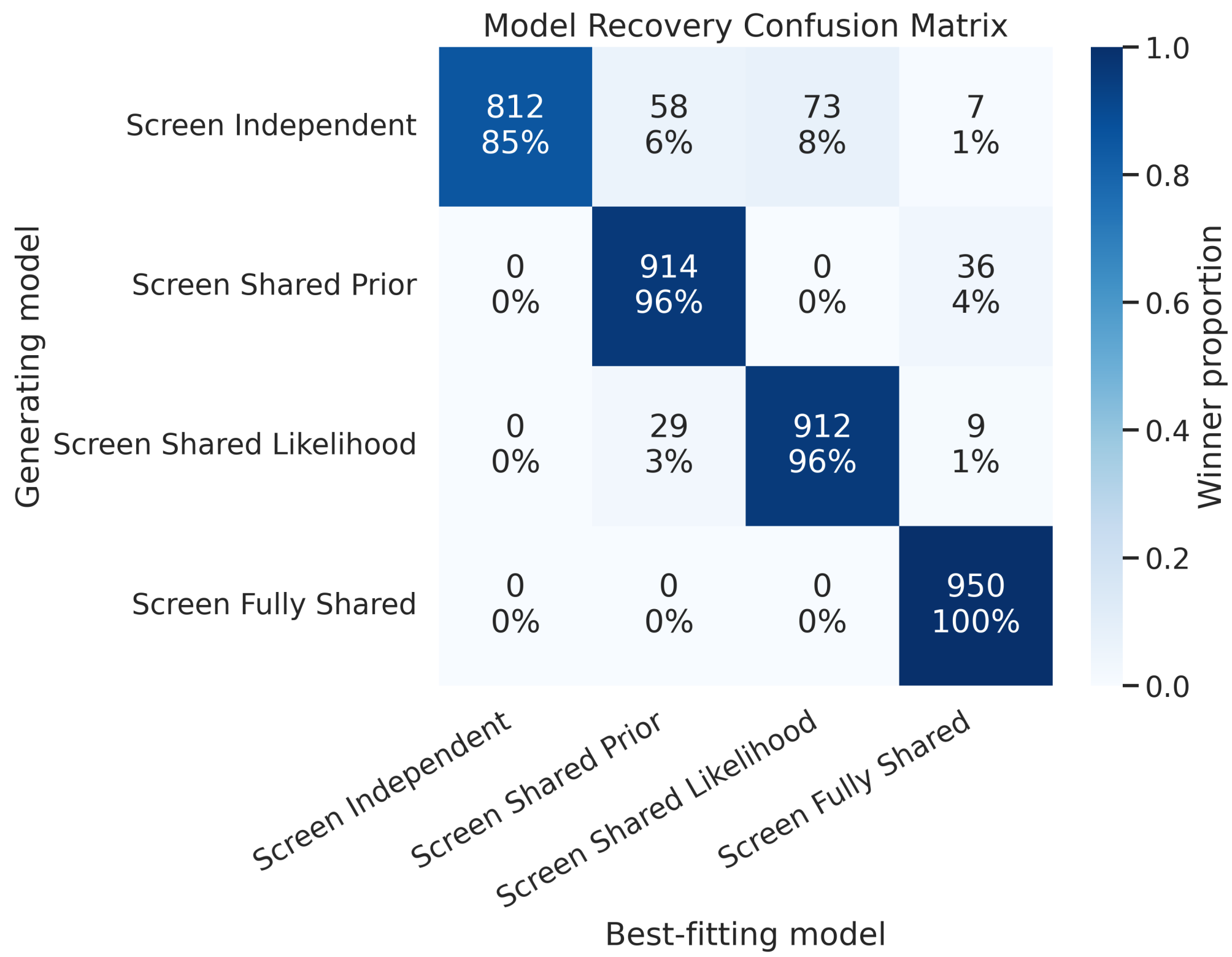

Fig C. Model recovery for the screen-space constrained model family. Rows indicate the generating model, and columns indicate the recovered best-fitting model. Entries show counts and percentages across simulated datasets. True-model recovery was high across the model family: 85.5% for the Independent Model, 96.2% for the Shared Prior Model, 96.0% for the Shared Likelihood Model, and 100% for the Fully Shared Model. Shared-model datasets were not spuriously classified as Independent, indicating that the empirical preference for modality-specific priors was not caused by a generic selection bias toward the most flexible model.
